# Can a doctor be too old to practice medicine?

**DOI:** 10.3389/frhs.2025.1521832

**Published:** 2025-08-25

**Authors:** Jonathan T. W. Au Eong

**Affiliations:** Lee Kong Chian School of Medicine, Nanyang Technological University, Singapore, Singapore

**Keywords:** medicine, ethics, safety, age, retirement

## Introduction

Recent articles on some doctors working in their 90s have resurfaced the complex issue of mandatory retirement for doctors (1–3). This question of timely retirement has been debated for many years and is not unique to the medical profession. Industries such as aviation and judicial law have mandatory retirement ages for public safety, acknowledging the detriment of aging on one's performance (4).

With life expectancy increasing, Singapore employees can only be asked to retire at age 64 from 2026, a progressive move towards raising the retirement age to 65 by 2030. As there is currently no mandatory retirement age for doctors in Singapore, how long a doctor chooses to practice is usually a personal choice. This is similar to large countries such as the United States (US), United Kingdom and Australia amongst others (5). Other countries such as China, India, and Russia have established retirement ages ranging from 55 to 65 for doctors (5).

There are a variety of reasons for the mandated retirement ages across countries. In some countries, public service doctors are classified similarly to other civil servants, resulting in blanket retirement ages across all civil service sectors. Mandatory retirement ages for public service doctors also ensures vacancies for new incoming medical graduates entering the system. System-based incompetencies may also hinder the application of competency-based retirement in some jurisdictions. In India and China for example, there are no requirements for license renewal by an independent body. Despite a 2011 resolution by the Medical Council of India requiring doctors to complete 30 h of Continuing Medical Education (CME) every five years to ensure re-registration, only nine of 26 state medical councils have made re-registration for license renewal mandatory, resulting in only 20% of India's doctors following CME rules (6).

Doctors are not immune to age-related physiological decline, leading to concerns that older doctors may compromise patient safety or even cause harm (4). In the spirit of beneficence and non-maleficence described by Beauchamp and Childress (7), patient safety in an older doctor's practice is of foremost consideration. Any doctor's continued practice should be in his/her patients’ best interest and should not lead to any harm.

This essay critically explores this ethical dilemma and examines ways to protect the public and prevent an erosion of trust in the profession.

## Age-related physiological decline

### Cognitive function

The healthy human brain undergoes significant changes with age, typically starting in the middle adult years with a decline in fluid intelligence, while crystallised intelligence remains stable (8). Fluid intelligence refers to reasoning and information processing capacity while crystallised intelligence is the accumulation of knowledge throughout life (9). Mean cognitive ability falls by 20% between the ages of 40 and 75 years in the general population (4), manifesting as declines in information processing (10, 11), selective and divided attention (12), and manual dexterity (13).

### Motor skills

Aging brings about a decline of motor skills (14) due to dysfunction of the neuromuscular, and central and peripheral nervous systems (15). This affects the ability to learn new movements by practice, integrate motor and cognitive tasks, and coordinate movement sequences (14).

### Special senses

Aging is associated with deterioration of certain special senses (10). Age-related hearing loss is the most prevalent sensory deficit in the elderly (16), resulting in high frequency hearing loss and poor speech recognition that negatively impacts cognitive, emotional, and social function (17). Age-related visual deterioration has also been well-documented, with declines in visual acuity from middle age (18). It is also associated with decreased light sensitivity and contrast, color discrimination, temporal sensitivity, motion perception, peripheral visual field sensitivity, and visual processing speed (19, 20).

## Potential effects of age-related physiological decline in an older doctor on patient care

Medical practice requires a combination of cognitive and physical domains to accurately diagnose and treat patients, and to carry out procedures. Aging and its associated physiological decline may affect a doctor's competency and hamper his/her ability to make sound judgements, thus compromising medical care. A systematic review found that older doctors possess less factual knowledge, are less likely to adhere to appropriate standards of care, and may have poorer patient outcomes compared to younger doctors (21). A US study on doctor age and outcomes in hospitalized older patients found that within the same hospital, patients treated by older doctors had a higher mortality than patients cared for by younger doctors, except for doctors treating high volumes of patients (22).

Advancing age results in difficulties in retrieving known information and learning new abilities (10). With new medical advances and increasing complexities in patient care, CME is widely accepted by the profession as critical to keeping up-to-date with new developments within the medical field (23, 24). Age-related cognitive decline may hamper the ability of a doctor to absorb and retain new information, hindering his/her ability to keep abreast with advancements in diagnosis, treatment protocols, and technologies.

## Arguments for allowing an older doctor to continue practicing

### Limitations of using age as a sole determinant of competency

To dismiss a doctor's right to practice solely based on his/her age is unjust and could raise potential questions about age discrimination. Importantly, the correlation between chronological and physiological age is highly variable (25) as the rate of age-related physiological decline varies between individuals (5, 9).

Furthermore, multiple factors besides age influence a doctor's competence to practice. These include:

1. Doctor factors such as intelligence, motivation for self-directed learning, and intentional practice to maintain proficiency.
2. Patient factors such as disease acuity and complexity.
3. Practice factors such as staffing and support systems (26).

For example, studies have found that failure to keep abreast with medical developments is more common amongst doctors in solo practice or with few peer interactions, regardless of age (27, 28). Personal health issues may also affect competency. It is thus imperative that each doctor be assessed individually and not be adjudged to be incompetent based solely on age.

### Positive qualities of older doctors

An older doctor's wealth of clinical experience given his/her number of years in practice must be acknowledged. Increased experience can provide greater efficiency in diagnostic skills involving pattern recognition (9). Furthermore, older doctors are more likely to be attributed with positive traits that are associated with good quality medical care and patient satisfaction (29), such as meticulousness, kindness, and willingness to listen (30).

### Negative impact of retirement of older doctors on patient care

Mandated retirement of older doctors at a set age can adversely impact patient care. Within general practice, the forced retirement of doctors may result in discontinuity of care through the loss of long-standing doctor–patient relationships (31). A systematic review found that doctor retirement has an unfavorable outcome on most patients, particularly those on long-term follow-up (32). Older patients with multiple co-morbidities and social care needs are at greatest risk of harm from care interruption and may seek healthcare in emergency departments instead of choosing a new doctor, resulting in fragmented care, poor information handover, and ultimately adverse clinical outcomes (31).

## Scarcity of manpower within the healthcare profession

Medical manpower forecasting is notoriously difficult, and the experience in many countries has seen many challenges. At a time when many parts of the world are grappling with a relative lack of doctors, this issue may be further exacerbated if inflexible, mandatory retirement policies for doctors are strictly enforced with no room for competency-based assessment.

## Special considerations

An important consideration to take into account when considering whether older doctors should be allowed to practice is setting and specialty. This is particularly pertinent in the fields of surgery and medical specialties which are procedural in nature. These fields require a combination of technical skills requiring dexterity and quick decision-making in an ever-changing environment where unpredictable mishaps may occur.

As discussed above, physiological aging and neurodegenerative disease have significant impacts on cognition and physicality, including aspects such as slowing of processing speed, executive cognitive function, and spatial orientation (31). Thus, older physicians may experience greater difficulty in transforming information to decisions in critical situations, quickly responding to stimuli or handling multiple and complex tasks simultaneously (31). This may pose a danger to patients and adversely affect patient outcomes. For example, a meta-analysis on the association between surgeon age and postoperative complications/mortality found that the mortality in patients undergoing surgery by older surgeons was 1.14 compared to those by middle-aged surgeons and 1.23 compared to those by younger surgeons (33). Anesthesiologists above the age of 65 have also been found to have double the risk of litigation compared to colleagues under the age of 51 (34). However, it must be acknowledged that there are other factors other than age that might contribute to higher litigation rates, such as higher patient volume and complexity of cases.

## Ensuring safe medical practice by older doctors

The above arguments support an older doctor being allowed to practice provided he/she is competent. A mandatory retirement age can be avoided through a combination of complementary measures to certify competence of older doctors. Competence to practice has historically been judged by one's peers, and this should be assessed individually, focusing on functional ability instead of chronological age. A fine balance between preserving patient safety and allowing older doctors to continue medical practice must be achieved.

Current safeguards of patient safety include:

1. Reactive assessment—doctors can be identified and evaluated after an error has occurred.
2. Self-assessment—in the spirit of the Hippocratic oath, doctors should be cognizant of their own competence and voluntarily restrict their own practice to areas they are comfortable in.
3. Peer reporting—in many jurisdictions including Singapore, doctors are obliged to report their colleagues if they have reasons to believe that their colleagues are so impaired in performance or fitness to practice that is clearly unsafe for them to continue managing patients (35).

However, these safeguards are intrinsically insufficient given that doctors are anecdotally reluctant to notify authorities about a colleague's declining performance (8), may lack insight due to cognitive decline (36) and may be resistant to self-reporting for fear of professional, societal, and legal sanctions (37, 38).

### Age-mandated assessment

A more proactive approach which involves standardized screening tests or peer evaluations beginning at a certain age to identify age-related performance issues before they result in patient harm would be helpful. Mandatory testing of cognitive function and manual dexterity for doctors beginning at age 75, where the increase in incidence of Alzheimer's disease is well-documented (5), and thereafter every few years could be established. Mandatory testing of cognitive function has been instituted in some countries like the US. In 2012, Stanford University Medical Centre introduced a compulsory biennial physical examination, cognitive assessment, and peer evaluation of clinical performance for all doctors starting at age 75 (5). Besides preventing possible patient harm, age-mandated assessments could potentially lead to identification of underlying medical conditions at an early, treatable stage, helping doctors remain in practice with treatment.

### Transitioning out of practice

For doctors who are deemed no longer able to practice safely, alternative roles such as teaching, mentoring, and administrative opportunities could allow older doctors to transition out of clinical practice while continuing to contribute to the medical community.

## Conclusion

In summary, it is important to balance an older doctor's continued ability to practice with the protection of patient welfare. Older doctors who demonstrate that they remain competent despite their age through appropriate assessments should be allowed to remain in practice in the right settings. Rather than imposing a mandatory retirement age, periodic evaluations of clinical ability may offer a more equitable and balanced approach to ensuring patient safety and protecting public trust in the profession.
